# Methionine synthesis and glycine betaine demethylation are intricately intertwined in cosmopolitan marine bacteria

**DOI:** 10.1073/pnas.2426167122

**Published:** 2025-09-16

**Authors:** Michaela A. Mausz, Andrew R. J. Murphy, Maria del Mar Aguilo-Ferretjans, Andrew Hitchcock, Mary Ann Moran, David J. Scanlan, Yin Chen, Ian D. E. A. Lidbury

**Affiliations:** ^a^School of Life Sciences, University of Warwick, Coventry CV4 7AL, United Kingdom; ^b^Molecular Microbiology—Biochemistry and Disease, School of Biosciences, University of Sheffield, Sheffield S10 2TN, United Kingdom; ^c^Plants, Photosynthesis and Soil, School of Biosciences, University of Sheffield, Sheffield S10 2TN, United Kingdom; ^d^Department of Marine Sciences, University of Georgia, Athens, GA 30602; ^e^School of Biosciences, University of Birmingham, Edgbaston B15 2TT, United Kingdom

**Keywords:** microbial oceanography, methylated metabolites, marine bacteria, glycine betaine, cobalamin-dependent methyltransferases

## Abstract

Across all domains of life, cobalamin-dependent methyltransferases have diversified to perform a range of crucial functions, such as methionine synthesis and the demethylation of various reduced nitrogen and sulfur compounds. These large modular enzymes typically possess three substrate-binding domains, two binding either the methyl donor or methyl acceptor, as well as a cobalamin-binding domain. Here, by challenging the current paradigm of glycine betaine (GBT) catabolism, we have identified a unique methyltransferase in aerobic environmental bacteria that has a dual function both as a methionine synthase and a GBT methyltransferase. Using the marine bacterium *Ruegeria pomeroyi* DSS-3 as a model, we demonstrate that a core cobalamin-binding domain (MtgC) and a bidirectional methyltransferase (MtgD) are essential for both methionine synthesis and GBT demethylation. MtgC is phylogenetically distinct from the cobalamin-binding domains of either the classical methionine synthase (MetH) or the GBT methyltransferases found in anaerobic bacteria and archaea. Across the global ocean, *mtgC* expression is frequently greater than previously known GBT catabolic pathways due to its occurrence in abundant cosmopolitan marine bacteria. Thus, we uncover a unique relationship between GBT catabolism and methionine synthesis in nature and identify a major route for N-osmolyte demethylation in the global ocean.

## Significance

Ocean productivity is driven by the interaction of phototrophic and heterotrophic microorganisms. Glycine betaine, which is an important osmolyte, nutrient, and energy source for marine microorganisms, is a key metabolite influencing these interactions. Demethylation of glycine betaine represents the first step of this metabolic pathway; however, the required molecular mechanisms have remained elusive. Here, we identify a pathway for glycine betaine demethylation, demonstrating its widespread occurrence and high activation across the global ocean. We reveal a multimeric cobalamin-dependent methyltransferase, recently identified as an alternative methionine synthase, has a dual function in glycine betaine demethylation. This modular enzyme may enhance the growth of cosmopolitan marine bacteria by enabling them to demethylate numerous phytoplankton-derived methylated compounds across the global ocean.

The flow of energy and organic matter in surface ocean waters represents a significant component of the global carbon cycle and a gateway for the deposition of atmospheric carbon dioxide (CO_2_) to the deep ocean, also known as the microbial carbon pump (1). This pump is driven by the vast number of ecological interactions occurring between marine microbes that inhabit these waters. Photosynthetic organisms fix CO_2_ and produce a plethora of dissolved organic matter (DOM) which enters the water column via leakage, active secretion, and cell death (protist grazing or vial lysis). The subsequent pool of DOM provides a rich source of carbon and energy for marine microbes and is primarily consumed by marine heterotrophic bacteria (1). The metabolic action of marine heterotrophic bacteria recycles key nutrients like ammonium and phosphate, releasing them from DOM (2–7). This action fuels surface waters and helps sustain photosynthetic microbes and their productivity (6). Several metabolites playing a key role in phototrophic–heterotrophic interactions have recently been identified including dimethylsulfoniopropionate (DMSP), 2,3-dihydroxypropane-1-sulfonate (DHPS), methylamines, methanol, and glycine betaine (GBT) (1, 8, 9). Heterotrophic catabolism of these molecules also influences the production of atmospheric trace gases (10–12).

GBT is ubiquitous in the marine environment (13–15). In seawater extracellular GBT concentrations are in the subnanomolar range (16) due to high affinity transport systems (17). With intracellular concentrations reaching the low to mid mM range in dense phytoplankton cultures (18, 19), GBT is among the most abundant intracellular metabolites in natural communities (15). GBT is produced, released, and taken up by various marine phototrophic and heterotrophic microbes, including microalgae and cyanobacteria (16, 20–23), serving as an osmolyte or nutrient for marine heterotrophic bacteria particularly from the Alpha- and Gammaproteobacteria groups (8, 16, 23). While it is well known that GBT is rapidly acquired from seawater for these functions (11), the genes and enzymes responsible for GBT catabolism in marine bacteria are poorly characterized (8). Bacteria and archaea have seemingly evolved various pathways to utilize exogenous GBT as a sole carbon and energy source (*SI Appendix*, Fig. S1). In *Pseudomonas* spp. and *Chromohalobacter salexigens,* a GBT monooxygenase, encoded by *gbcAB*, is responsible for the demethylation of GBT, producing dimethylglycine (DMG) and formaldehyde (24, 25). Conversely, in the plant symbiont *Sinorhizobium meliloti*, a betaine-homocysteine methyltransferase (Bhmt, named Bmt in ref. 26), similar to human Bhmt, was identified as having a role in GBT catabolism (26). However, this study also revealed the presence of another GBT catabolic system that was operational upon the addition of methionine to the culture medium. In marine systems, SAR11 clade bacteria such as *Candidatus* Pelagibacter ubique can oxidize GBT to CO_2_, generating energy that supplements their heterotrophic growth (27) and can even grow on GBT as a glycine substitute (17), relieving the unusual glycine auxotrophy of this bacterium (28). Although not experimentally validated, SAR11 clade bacteria possess a Bhmt more similar to the human enzyme than the homolog identified in *S. meliloti*, with the latter lacking residues required for GBT binding [*SI Appendix*, Fig. S2 and (26)].

**Fig. 1.**
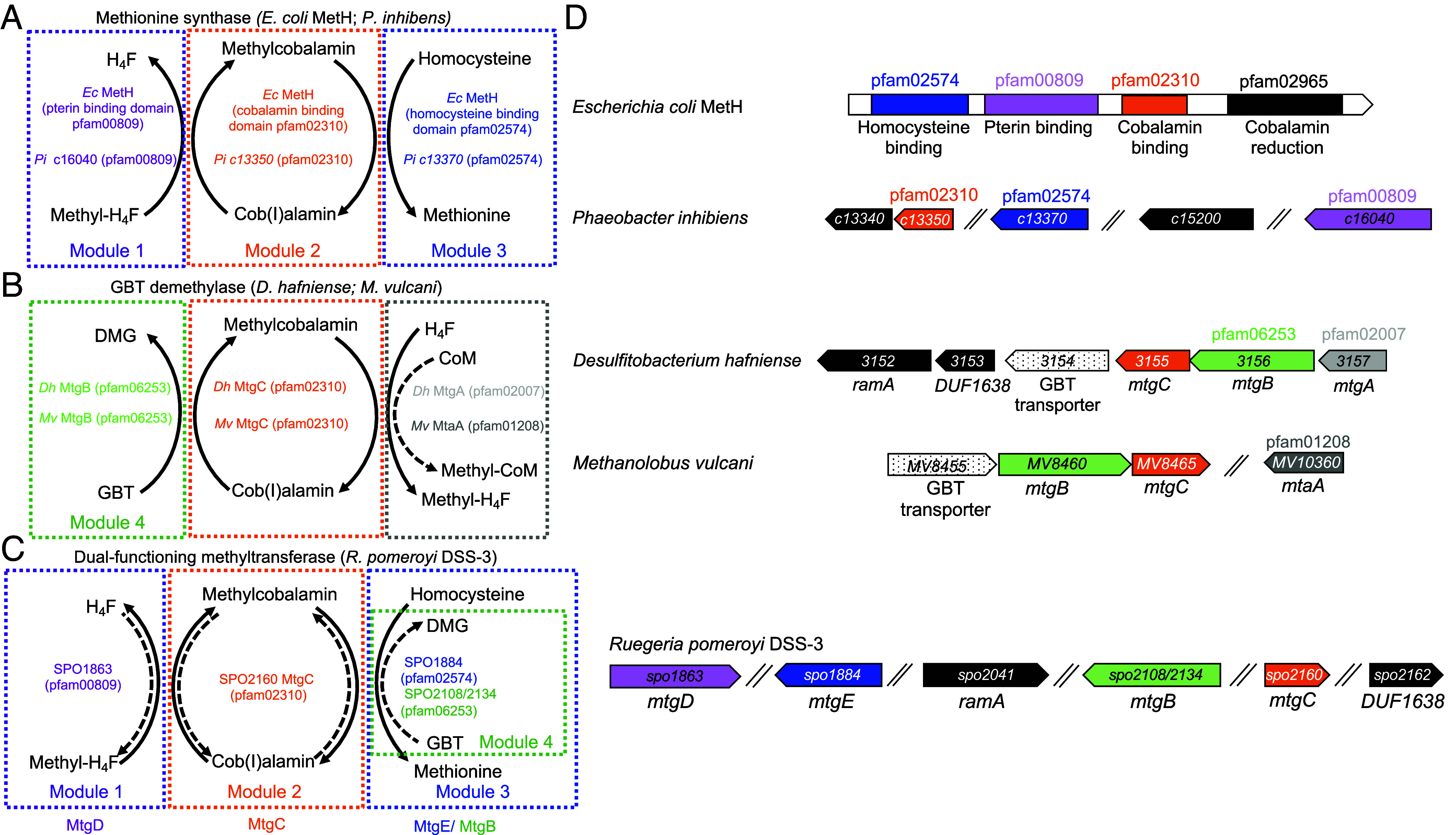
Schematic modules of cobalamin-dependent methyltransferases in methionine synthesis and glycine betaine demethylation. (*A*) Modular composition of methionine synthase of *E. coli* MetH and *P. inhibens*, composed of a tetrahydrofolate binding domain (pfam00809, module 1), a cobalamin-binding domain (pfam02310, module 2), and a homocysteine-binding domain (pfam02574, module 3). (*B*) Modular composition of the GBT demethylase in methanogenic and nonmethanogenic microorganisms. GBT binding is mediated by a pfam06253 methyltransferase (module 4) which subsequently donates the methyl group to the cobalamin-binding protein (MgtC, pfam02310). Methylated cobalamin subsequently donates the methyl group to either tetrahydrofolate (nonmethanogens) or coenzyme M (CoM) (methanogens). Note that the cobalamin-binding domains of MetH and MtgC share the same pfam domain (pfam02310). (*C*) Proposed model connecting GBT demethylation (modules 1, 2, 4) and methionine synthesis (modules 1, 2, 3) through MtgC in *R. pomeroyi* DSS-3. (*D*) Genetic loci associated with either GBT methyltransferase or methionine synthase. Abbreviations: *Dh, Desulfitobacterium hafniense; Ec, Escherichia coli*; *Mv, Methanolobus vulcani; Pi, Phaeobacter inhibens; ramA*, corrinoid protein reductive activase.

We have previously shown that members of the marine roseobacter group, including the model species *Ruegeria pomeroyi*, can utilize GBT and its precursor choline as sole carbon and nitrogen sources (8). Carnitine, another nitrogen containing-osmolyte (N-osmolyte), may also be converted to GBT in these bacteria (29). While the genes for choline metabolism have been experimentally validated (8), the molecular mechanism governing GBT demethylation to DMG in *R. pomeroyi* and marine bacteria in general, except for the predicted gene in *Pelagibacter* (27), remains unknown. In anoxic systems, GBT reduction to trimethylamine was traditionally thought to be the main pathway responsible for the removal of this osmolyte. However, in the anaerobic Gram-positive bacterium *Desulfitobacterium hafniense,* a GBT:cobalamin methyltransferase and a methyl-cobalamin:tetrahydrofolate (H_4_F) methyltransferase were identified and induced during growth on GBT, along with a corrinoid-binding protein (30). It was proposed that these three proteins constitute a cobalamin-dependent methyltransferase with the corresponding genes named: *mtgB*, *mtgA*, and *mtgC*, respectively (Fig. 1). Several *mtgB* homologs were found in marine bacteria, including members of the cosmopolitan marine roseobacter group, suggesting methylated compounds such as GBT may be demethylated via similar mechanisms (30).

Methyltransferases using cobalamin (also known as B_12_) or related corrinoid analogs as a cofactor are common in all domains of life (31) and perform various functions (Fig. 1). In anaerobic bacteria and archaea, this group of enzymes enables energy generation from the demethylation of methylamines and similar compounds (32, 33), while in eukaryotes, they function solely as a methionine synthase. A cobalamin-dependent methyltransferase responsible for methionine synthesis from homocysteine is also common among various aerobic bacteria. The bacterial cobalamin-dependent methionine synthase (MetH) is found in a wide range of taxa. MetH is a modular protein with three substrate-binding domains (module 1, 2, and 3) and a cobalamin-reduction domain (pfam02965) (Fig. 1). The marine bacterium *Phaeobacter inhibens* lacks *metH* but harbors three separate genes (PGA1_c13370, PGA1_c13350, and PGA1_c16040) each encoding one of the three substrate-binding MetH domain functions (34), together with two genes (PGA1_c15200 and PGA1_c13340) hypothesized to be involved in the reductive recharging of the cobalamin cofactor (Fig. 1*D*) (30, 34, 35). Thus, it appears that enzymes composed of modular components appear common in methionine synthesis and GBT demethylation, both requiring a cobalamin-binding domain protein (pfam02310).

Here, we combine proteomics and bacterial genetics to reveal a holistic model for GBT metabolism and methionine synthesis in marine bacteria lacking *metH*, using *R. pomeroyi* DSS-3 as the model (Fig. 1*C*). We demonstrate that a unique methyltransferase has evolved to convergently function as a dual methionine synthase-GBT methyltransferase, thus uncovering an unexpected connection between GBT demethylation and methionine synthesis in these organisms. Using these new molecular markers for GBT demethylation, integrated metaomics revealed that diverse cosmopolitan oligotrophic and copiotrophic Pseudomonadota (formally known as Proteobacteria) significantly contribute toward GBT consumption across the global ocean.

## Results

### Proteomics Reveals Growth on Glycine Betaine Requires a Unique Methyltransferase.

**Fig. 2.**
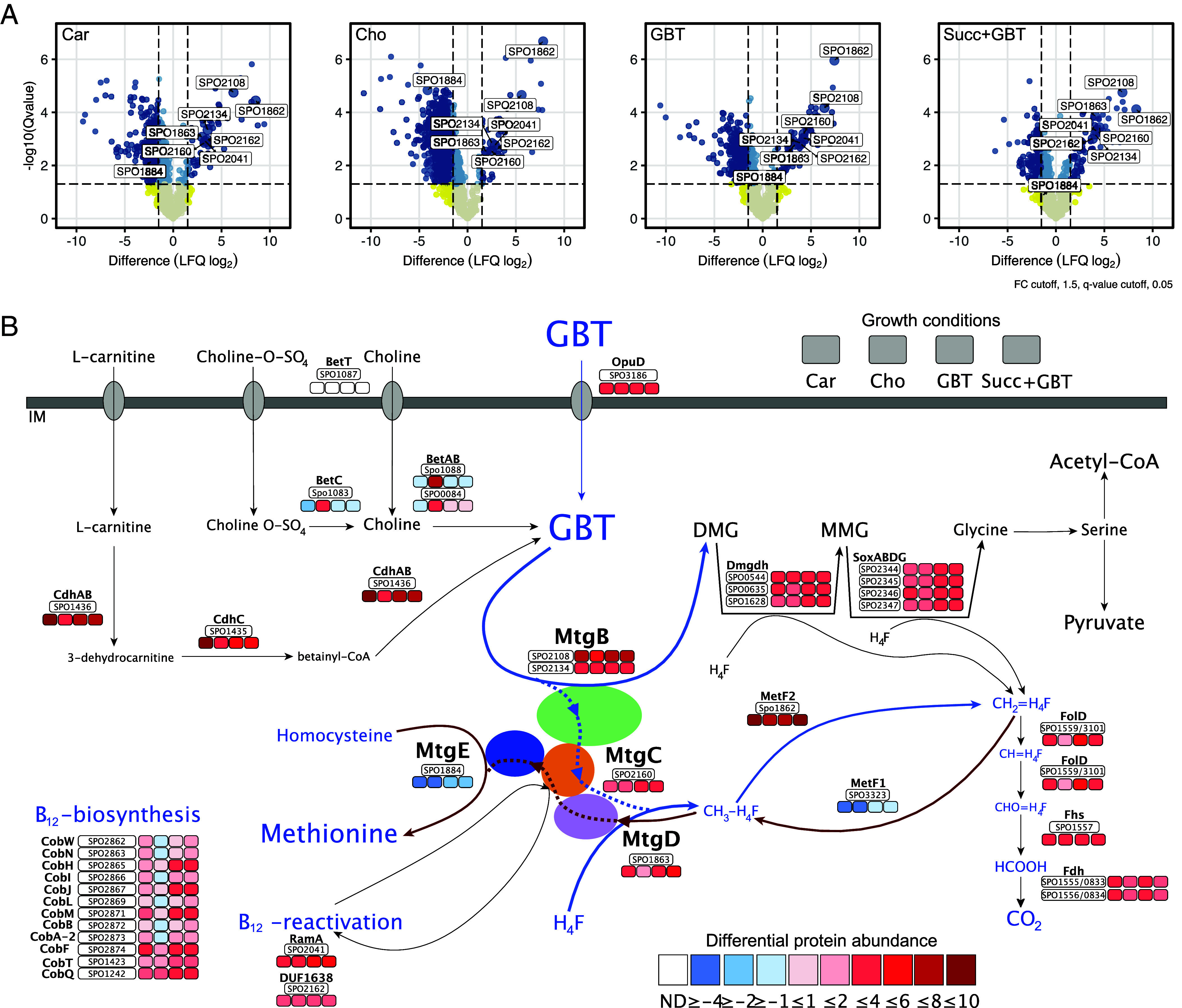
Proteomic assessment of *R. pomeroyi* grown on glycine betaine and other methylated compounds. (*A*) Whole-cell protein extracts were prepared from cells grown on succinate (Succ) and ammonium (control) or GBT, choline (Cho), or carnitine (Car) as the sole carbon source. A fifth treatment with Succ as the carbon source and GBT as the sole nitrogen source was also analyzed. The mean (n = 3) difference in LFQ*^Log2^* intensity values between control (succ+ammonium) and N-osmolyte treatments is presented. (*B*) Scheme of GBT catabolism where MtgB (green) donates a methyl group to MtgC: Cobalamin (orange) and MtgD (purple) transfers the methyl group onto tetrahydrofolate (H_4_F), depicted by a blue dotted line. We hypothesize that MtgD also works in reverse (red dotted line) to transfer the methyl group to MtgC: cobalamin followed by transfer to MtgE (blue), which converts homocysteine to methionine. IM, inner membrane; ND, not detected.

To identify the key proteins involved in GBT utilization, we performed comparative proteomics on *R. pomeroyi* DSS-3 cells grown on either succinate (control), carnitine, choline, or GBT as the sole C source (Fig. 2 and Dataset S1). A condition combining succinate as the C source and GBT as the N source was also established. A total of 1,750 proteins were detected across all conditions, of which between 37 (choline-grown) and 69 (GBT-grown) proteins showed increased synthesis (FDR corrected *P* < 0.05, log_2_ fold change>2) during growth on N-osmolytes. These included proteins related to methylotrophy, such as the tetrahydrofolate C_1_ oxidation pathway and serine cycle, as well as L-serine-ammonia lyase (SdaA) that converts serine into pyruvate and ammonium (*SI Appendix*, Fig. S3). The latter represented some of the most differentially synthesized proteins in all N-osmolyte conditions. In addition, the synthesis of proteins required for the transformation of carnitine (CdhABC) and choline (BetABC) to GBT, predicted dimethylglycine and sarcosine (monomethylglycine, MMG) demethylation steps, and a predicted BCCT-type GBT transporter (OpuD) was also increased.

N-osmolyte-grown *R. pomeroyi* DSS-3 cells also synthesized several proteins encoded by separate ORFs with homology to those identified as a split methionine synthase in *P. inhibens* (34) and those encoding the three-subunit GBT demethylase in *D. hafniense* (30) and *Methanolobus vulcani* (33). These include a cobalamin-binding protein (encoded by SPO2160, MtgC) and a pterin-binding protein (encoded by SPO1863, which we term MtgD). MtgD and MtgC are homologous to the pterin-binding subunit (module 1) and cobalamin-binding subunit (module 2) of the split methionine synthase in *P. inhibens* (Fig. 1). The homocysteine binding subunit (module 3) of methionine synthase (PGA1_c13370 in *P. inhibens* (34), which we term MtgE) is encoded by SPO1884 in *R. pomeroyi* DSS-3 and was slightly less abundant during growth on N-osmolytes, consistent with constitutive production and an essential role in methionine synthesis during growth in minimal medium lacking exogenous methionine. MtgE is also homologous to the predicted Bhmt in *S. meliloti* (25) (*SI Appendix*, Fig. S2). Two proteins (SPO2108, SPO2134) containing a pfam06253 domain that is known to be a bona fide MtgB involved in GBT demethylation in *D. hafniense* (30) and *M. vulcani* (33) also increased in relative abundance (Fig. 2). Downstream of *mtgD* is a paralog of *metF* (SPO3323) that we name *metF2* (SPO1862). MetF2 was the most differentially synthesized protein during growth on all three N-osmolytes, suggesting that this enzyme oxidizes methyl-H_4_F produced by MtgBCD into 5,10-methylene-H_4_F, serving as the entry point for H_4_F-linked C1 oxidation (Fig. 2), similar to the methyl group oxidation of trimethylamine and trimethylamine *N*-oxide (9). Proteins associated with the serine cycle, as well as L-serine-ammonia lyase (SdaA) that converts serine into pyruvate and ammonium, were also differentially synthesized in greater relative abundance (*SI Appendix*, Fig. S3 and Dataset S1).

**Fig. 3.**
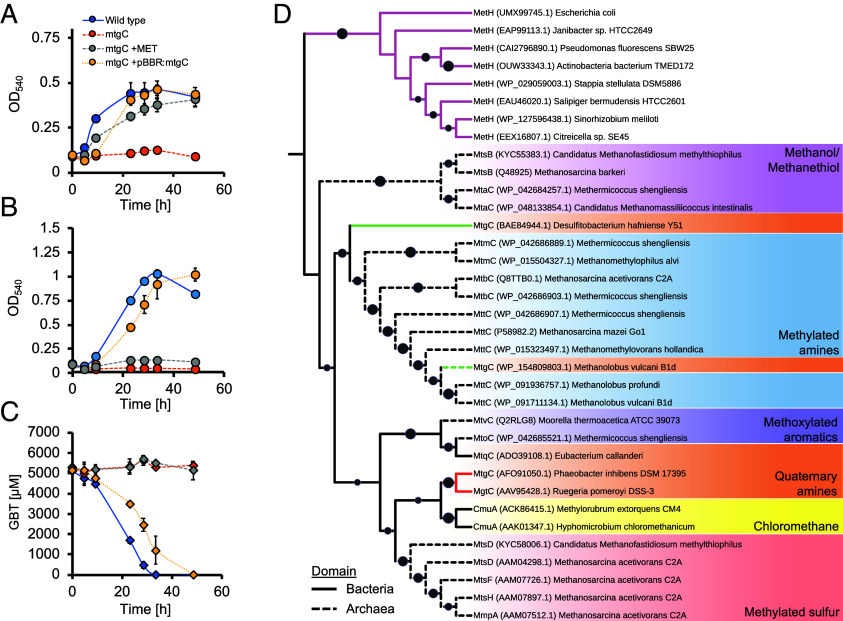
Functional validation and phylogeny of MtgC in *R. pomeroyi* DSS-3. The *R. pomeroyi* DSS-3 wild type, *mtgC* mutant and complemented *mtgC* mutant (*mtgC* +pBBR-*mtgC*) were grown in minimal medium with succinate and ammonium as the sole carbon and nitrogen sources (*A*) or GBT as the sole carbon source (*B*). Quantification of GBT in the culture supernatant is presented in (*C*). For the *mtgC* mutant, additional cultures supplemented with methionine (+MET) were also established. Results are the mean of triplicate cultures and error bars denote the SD. (*D*) MtgC homologs in *R. pomeroyi* DSS-3 and *P. inhibens* (red) were aligned with other cobalamin-binding domains/proteins associated with various cobalamin-dependent methyltransferases. Phylogenetic reconstruction was performed using the maximum likelihood method including the domain found in classical MetH (pink branches) and MtgC characterized in the Gram-positive bacterium *D. hafniense* and the methanogenic archaeon *M. vulcani* (green branches). Bootstrap values >51% are given on branches, scaled so higher values equal larger circles.

In agreement with MtgC predicted to be cobalamin-dependent, like GBT demethylase in *D. hafniense* (30), proteins for cobalamin synthesis were more abundant during growth on methylated compounds relative to the succinate control (Fig. 2 and Dataset S1). Likewise, the proteins encoded by SPO2041 (RamA) and SPO2162 (DUF1638), predicted to enable the reductive recharging of cobalamin (cob[II]alamin > cob[I]alamin) in *P. inhibens*, *D. hafniense*, and *M. vulcani* (30, 33, 34) (Figs. 2*B* and 3), were also more abundant when the cells were grown on N-osmolytes.

### MtgC and MtgD Are Essential for Methionine Synthesis and GBT Demethylation.

**Fig. 4.**
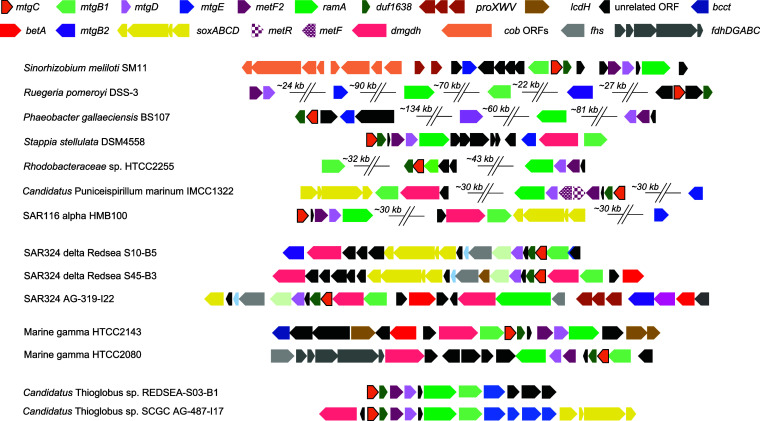
The genomic organization of GBT demethylation and methionine synthase genes in phylogenetically distinct marine bacteria. The genetic neighborhoods of *mtgC* homologs were analyzed for the presence of associated cobalamin-dependent methyltransferase subunits and GBT metabolism genes. ORFs with related metabolic functions are also highlighted.

Given the high sequence identity of MtgC with the cobalamin-binding domain protein (PGA1_c13350) essential for methionine synthesis in *P. inhibens*, we hypothesized that this protein may have a dual role in both GBT demethylation and methionine synthesis. To assess whether SPO2160 does indeed encode a dual functioning protein (Fig. 1*B*), we generated a knockout mutant (Δ*mtgC::Gm*) in *R. pomeroyi* DSS-3. While growth of Δ*mtgC::Gm* was unaffected in rich medium (*SI Appendix*, Fig. S4), in minimal medium lacking methionine growth was arrested, suggesting this mutant was a methionine auxotroph (Fig. 3*A*). Similarly, a SPO1863 (*mtgD*) deletion also displayed methionine auxotrophy (*SI Appendix*, Fig. S5). Addition of methionine (0.1 mM) to the minimal medium restored growth of the *mtgC* and *mtgD* mutants on succinate as the sole carbon source. Neither growth on GBT as the sole carbon source (Fig. 3*B*) nor GBT consumption (Fig. 3*C*) could be rescued by methionine addition, demonstrating that *mtgC* encodes a core subunit for both the split MetH and GBT methyltransferase. Growth of the *mtgD* mutant on GBT could also not be restored upon methionine addition suggesting MtgD also functions bidirectionally (*SI Appendix*, Fig. S5). Complementation of Δ*mtgC::Gm* with a plasmid-encoded native *mtgC* reversed methionine auxotrophy and restored GBT catabolism and thus growth on this substrate as a sole carbon source, confirming the dual functionality of *mtgC* (Fig. 4 *A*–*C*). Based on these genetic and proteomic data, we propose a model for GBT demethylation in aerobic environmental bacteria that is intricately linked with methionine synthesis (Fig. 1*C*).

Phylogenetic placement of MtgC from *R. pomeroyi* DSS-3 and *P. inhibens* with all other corrinoid-binding domains of diverse archaeal and bacterial methyltransferases revealed a distinct origin (Fig. 3*D*). The cobalamin-binding domain of the classical multidomain MetH groups outside all other split methyltransferases. MtgC found in the marine roseobacter group is also distinct from the cobalamin-binding subunit of the GBT methyltransferase in the anaerobe *D. hafniense* (30) (Fig. 3*D*) and is closely related to the chloromethane methyltransferase (CmuA) found in Alphaproteobacteria.

### *mtgC* Is Present in Phylogenetically Diverse Marine Bacteria and Colocated with GBT Metabolism Genes.

To better understand the distribution of *mtgC* in marine ecosystems, we screened the genomes of various pelagic marine bacteria either retrieved from cultivated isolates, single-cell amplified genomes (SAGs), or metagenome-assembled genomes (MAGs). Homologs of *mtgC* were found in phylogenetically diverse groups of cosmopolitan pelagic marine bacteria related to Alpha-, Delta-, and Gammaproteobacteria, including the SAR116, SAR324, and SUP05 clades, respectively. We next scrutinized over 600 genomes of marine bacteria and found ~70% (108 out of 156) of genomes harboring genes for MtgBCD lacked the gene encoding the classical MetH. Conversely, only 28 out of 239 genomes harboring *metH* encode genes for MtgBCD. Genomes possessing both *metH* and *mtgBCD* were typically restricted to Hyphomicrobiales, the SAR324 cluster, and certain genera of Alphaproteobacteria (Dataset S2). The canonical *metE* was absent from most genomes harboring MtgBCD genes (Dataset S2). *Citreicella* sp. SE45 and other bacteria possessing the GBT monooxygenase (GbcAB), but not MtgBCDE, possessed the canonical MetH. These data suggest that MtgBCD found in phylogenetically distinct marine bacteria has evolved independently from MetH and the MtgBCD found in methanogens. The possession of this dual-functioning methyltransferase may have also enabled the subsequent loss of MetH, as supported by its absence in most MtgBCD-harboring bacteria while also significantly broadening the known requirement for cobalamin across the oceans.

Unlike *R. pomeroyi* DSS-3 and *P. inhibens*, ORFs required for GBT demethylation, including those required for cobalamin reactivation, C_1_ oxidation of methyl groups, and subsequent demethylation steps were frequently colocated on the genome and even found in operons (Fig. 4). In *S. meliloti*, ORFs encoding MtgBCDE, the two reductive reaction modules, and the cobalamin biosynthesis operon were all colocalized. In cosmopolitan marine Alpha-, Gamma-, and Deltaproteobacteria, genes encoding the subsequent demethylation steps of GBT were often found near *mtgBCD*, however, *mtgE* was not (Fig. 4). No MtgC homologs were encoded in Pelagibacteraceae genomes, consistent with the presence of a gene encoding a Bhmt homolog that shows greater similarity to the bona fide human Bhmt (*SI Appendix*, Fig. S2). SAR11 lack MetE, MetH, and the split methionine synthase (MtgBCDE). Hence, they rely on either exogenous methionine, DMSP (36), or presumably GBT (Bhmt) (27) for methionine synthesis.

### Genes Encoding MtgBCD Are Highly Expressed in the Global Ocean.

**Fig. 5.**
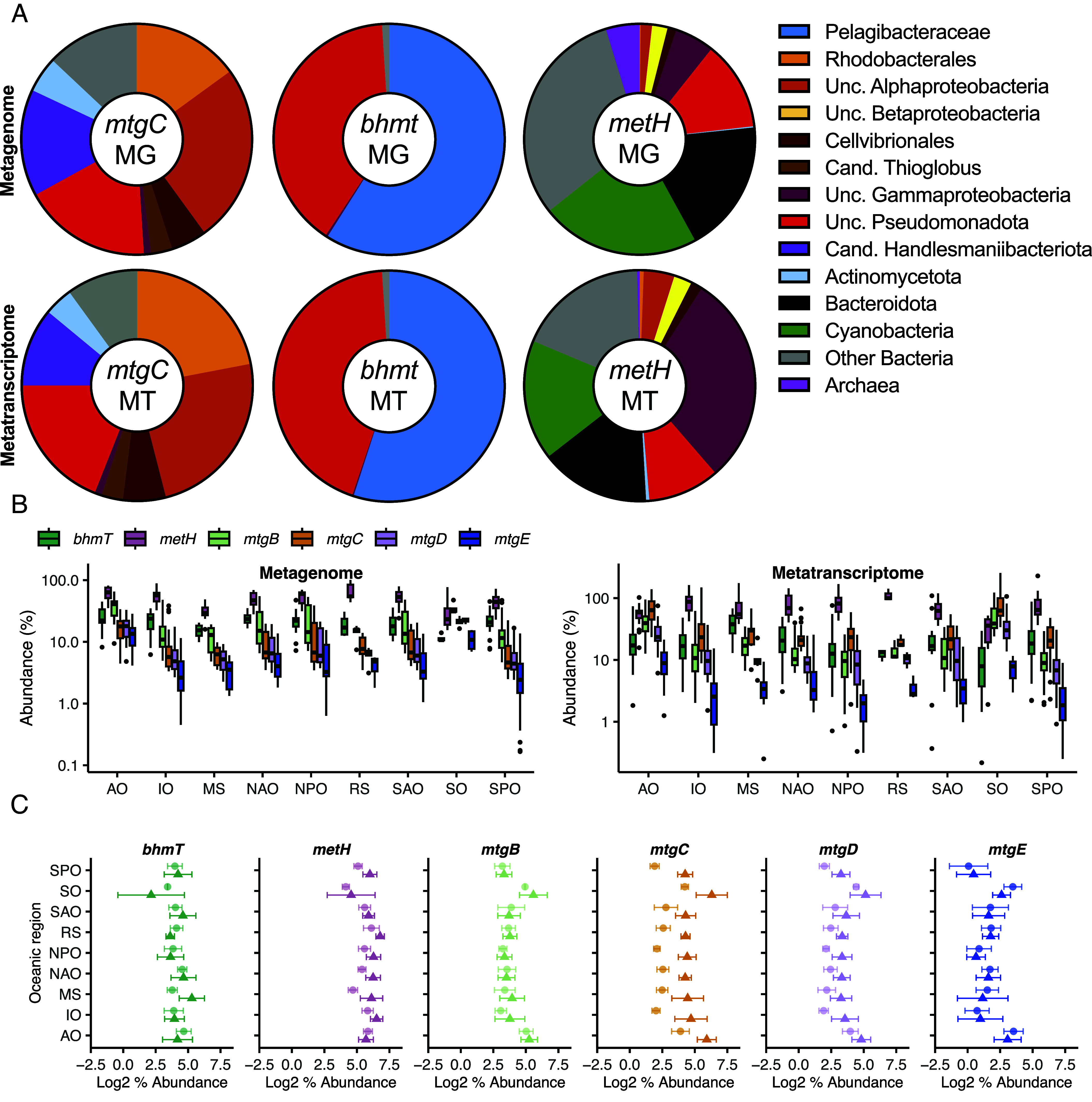
Comparison of *bhmt, metH,* and *mtgBCDE* distribution and expression in the global ocean. (*A*–*C*) The ocean gene atlas (32) was queried for the presence of *mtgC, bhmt,* and *metH* homologs and their overall diversity, gene, and transcript abundances were calculated (*A*). Gene and transcript abundances for *bhmt*, *metH,* and *mtgBCDE* were also determined for each oceanic region (*B*). A direct comparison of gene (circles) and transcript (triangles) abundance (Log2% abundance) within each oceanic region was also performed (*C*), demonstrating greater relative transcriptional activation of *mtgBCD* compared with *mtgE*. For % abundance of genes or transcripts, the number of mapped reads were normalized to the mapped reads of 10 single-copy core housekeeping genes as previously described (3). Abbreviations; AO, Arctic Ocean; RS, Red Sea; IO, Indian Ocean; MS, Mediterranean Sea; SPO, South Pacific Ocean; NPO, North Pacific Ocean; SO, Southern Ocean; SAO, South Atlantic Ocean; NAO, North Atlantic Ocean.

Given the occurrence of *mtgBCDE* in diverse pelagic marine bacteria, we scrutinized the TARA Oceans dataset using the Ocean Gene Atlas (OGA) portal. For comparison, the distribution, abundance, expression, and diversity of *mtgC* and the SAR11 clade *bhmt*, predicted to be essential for GBT demethylation were determined. In agreement with their limited occurrence in marine bacteria (Dataset S2), homologs of *gbcAB* (GBT monooxygenase) were low abundance and omitted from further analyses. While *bhmt* was predominantly found in pelagic marine Alphaproteobacteria, primarily Pelagibacteraceae, only 37% of total *mtgC* reads were assigned to Alphaproteobacteria, including Rhodobacteraceae (Fig. 5*A*). In contrast, *metH* diversity was dominated by Bacteriodota and Cyanobacteria that also were significant contributors to total *metH* expression, along with Gammmaprotoebacteria. Unlike *bhmt*, 10% of *mtgC* reads were assigned to the marine Gammaproteobacteria, including *Candidatus* Thioglobus, a member of the SUP05 clade. Almost 30% of reads were assigned to taxa outside of the Pseudomonadota phylum, including 5% classified as Actinomycetota. Consistent with its occurrence in SAR11 clade bacteria, *bhmt* was more abundant than *mtgC* across all oceanic sampling sites except for the Southern Ocean, being present in 10 to 30% of bacteria (Fig. 5*B*). In contrast, *mtgC* expression was equal to or greater than *bhmt* across all oceanic regions with the greatest *mtgC* expression occurring in regions where *bhmt* was lowest (Fig. 5 *B* and *C*). *metH* was the most abundant of the three gene markers across the ocean and only in polar regions was it transcribed less than *mtgC*, where expression of the latter gene was most pronounced toward higher latitudes (*SI Appendix*, Fig. S6). At sites recording the greatest total *mtgC* expression, the percentage of transcripts related to Rhodobacteraceae and Gammaproteobacteria was higher than at sites with lower total *mtgC* expression (*SI Appendix*, Fig. S7) At sites with lower *mtgC* expression levels, the contribution of *Candidatus* Handelsmaniibacteriota, unclassified Alphaproteobacteria, and unclassified Pseudomonadota increased relative to Rhodobacteraceae and Gammaproteobacteria.

To further infer the functional role of *mtgC* in marine bacteria, we also scrutinized the OGA for *mtgB, mtgD,* and *mtgE*. Abundance profiles of all three genes were comparable across the global ocean and consistent with that of *mtgC*. Gene expression profiles for *mtgB* and *mtgD* typically matched those of *mtgC* and were comparable to our proteomics data of GBT-grown *R. pomeroyi* DSS-3 cells, whereby MtgBCD were synthesized more than MtgE. These data suggest the GBT demethylation pathway is activated across the global ocean.

## Discussion

GBT is a common osmolyte in nature (16, 37, 38) and an important nutrient and energy source for environmental (20, 25–27, 30) and microalgae-associated bacteria (8). However, the molecular markers underpinning GBT utilization remain poorly resolved, particularly in marine ecosystems (8), impairing our ability to investigate in situ cycling at scale or specific co-culture studies. Here, we identified a unique multisubunit enzyme related to cobalamin-dependent methyltransferases involved in both GBT demethylation and methionine biosynthesis. Molecular characterization of GBT utilization genes revealed the occurrence of *mtgBCD* in cosmopolitan marine pelagic Alpha-, Gamma-, and Deltaproteobacteria, expanding the diversity of known GBT utilizers and providing an explanation for their proposed contribution to GBT consumption in seawater, based on molecular markers for DMG and MMG demethylation (16, 20).

Methylated N-osmolytes provide supplementary energy sources for marine bacteria and can help shorten their response to organic matter inputs and increase their viability during periods of starvation (6, 8, 39, 40). N-osmolyte turnover for supplementary energy generation results in the remineralization of ammonium providing a mechanism that facilitates the recycling of key inorganic nutrients which influences ocean biogeochemistry through stimulating phytoplankton production (6, 8, 40). While radiotracer incubation studies predict that a large fraction of GBT is retained as an osmolyte (20, 21, 23), our metatranscriptomics analysis shows the induction of genes involved in GBT demethylation across large parts of the ocean. This high level of *mtgBCD* transcription therefore suggests that GBT catabolism may mediate in situ heterotroph–phototroph interactions to support primary production via the release of ammonium (6–9, 40).

Although marine phototrophs are thought to be the major source of oceanic GBT, direct evidence for bacteria utilizing GBT produced by an algal partner during co-culture has been lacking (22). Eukaryotic microalgae–bacteria interactions were recently investigated using transcriptomics in a model system containing *Emiliana huxleyi* (coccolithophore) and *P. inhibens* (Rhodobacteraceae). In this study, upregulation of multiple genes required for methyl group metabolism were identified in co-culture versus monoculture (39). The homologs of these genes matched those highly abundant in N-osmolyte-grown *R. pomeroyi* cells (Fig. 2). This suggests that GBT is a key currency for metabolic exchange between phototrophs and heterotrophs—a process known to stabilize phototroph–heterotroph interactions under laboratory conditions (7). This includes the upregulation of *mtgBCD* and associated reductive reactivation of cobalamin genes, *opuD,* genes for the subsequent demethylation steps and genes for H_4_F -linked C_1_ oxidation.

MtgBCD represents an example of these modular enzymes operating in aerobic bacteria for GBT demethylation. Except for MetH, cobalamin-dependent methyltransferases are typically associated with bacteria and archaea inhabiting anoxic conditions (30, 31, 41), with GBT-specific and quaternary amine-specific cobalamin-dependent methyltransferases characterized in anaerobic bacteria (30, 42, 43). Our phylogenetic analyses revealed that the cobalamin-binding core subunit MtgC did not directly evolve from the classical MetH (28) nor the MtgC found in Gram-positive sulfur-reducing bacteria (30) or methanogenic archaea (33). Hence, the split methionine synthase previously identified in *P. inhibens* (34), the subunits of which closely resemble those identified here, have not simply separated from the classical MetH, but have separately evolved this function.

Our work also closes the long-standing knowledge gap on the environmental distribution of bacterial GBT catabolism and reconciles inconsistencies with previous studies reporting the molecular mechanisms governing GBT demethylation in aerobic bacteria that lack the dioxygenase GbcAB (24–26) but are still capable of utilizing this osmolyte as a nutrient and energy source (8, 27). We propose that only SAR11 bacteria, which lack MetH, MtgBCDE, and the cobalamin-independent methionine synthase MetE, use a single enzyme (Bhmt) to couple GBT demethylation to methionine synthesis (Fig. 1 and 20, 27). The nitrogen-fixing terrestrial alphaproteobacterium *S. meliloti* is also thought to synthesize Bhmt for direct coupling of GBT demethylation and methionine synthesis (26); however, two key observations presented in the Barra et al. (26) study suggested the story had not yet been fully resolved. First, GBT down-regulated *metH* expression, resulting in significant growth inhibition of the *bhmt* mutant during growth on other carbon sources without methionine supplementation. Second, supplementing methionine to the *bhmt* mutant enabled growth on GBT at levels comparable to the wild type with the authors noting that a distinct GBT demethylating pathway must exist. These data are consistent with our model for GBT demethylation via this unique multimeric enzyme, whereby the core MtgC interconnects methionine synthesis and GBT demethylation through interactions with MtgBCDE. Hence, in *S. meliloti, bhmt* is not a singular enzyme that directly couples GBT demethylation to methionine synthesis, rather it is homologous to *mtgE* in *R. pomeroyi* DSS-3 and *P. inhibens* functioning as a module on a multimeric enzyme, the latter experimentally proven to be essential for methionine biosynthesis (34). This would explain why only the SAR11-type Bhmt, and not *S. meliloti* and other marine bacterial homologs, retains the residues essential for GBT binding identified in human Bhmt. Our model therefore explains why GBT catabolism is unaffected in the *S. meliloti bhmt* mutant when supplemented with methionine (26), demonstrating that this gene (encoding MtgE) is not essential for GBT demethylation. This observation is consistent with our proteomics data for GBT-grown cells, whereby the relative abundance of MtgE decreases, unlike MtgBCD which all increase.

The homology of MtgC with other known cobalamin-dependent enzymes and the induction of proteins required for cobalamin synthesis, and its reductive reactivation, strongly suggests this enzyme requires cobalamin, further connecting the web of various metabolites and interactions in the global ocean (1). MtgBCD is present in genome-streamlined pelagic roseobacters that are B_12_ auxotrophs, losing cobalamin-synthesis genes during genome reduction, but subsequently acquiring a “gain of function” cobalamin transporter to “outsource” anabolism (44). It is tempting to speculate that the ecological benefits of GBT metabolism contributed to the fixation of the cobalamin uptake system, hence allowing them to expand their metabolic niche and capitalize on phytoplankton-derived nutrients. Bacteria, which are usually regarded as a source of cobalamin (45), also acted as a considerable sink taking up this vitamin at similar rates to eukaryotic microalgae in the Southern Ocean (46). Such high uptake could therefore be explained by MtgBCDE synthesis in bacteria lacking the *cob* operon, although this remains to be experimentally tested.

In addition to the canonical MetE, several cobalamin-independent methionine synthases resembling a truncated C-terminal MetE, named core-MetE and MetE-fusion, were recently discovered in anaerobic bacteria, archaea (47), and eukaryotic microalgae (48) (*SI Appendix*, Fig. S8). Homologs of the canonical MetE were only found in a few marine copiotrophs that typically also possess MetH, for example, Hyphomicrobiales and Vibrionales (Dataset S2). However, homologs of core-MetE proteins, which harbor the four conserved residues (Cys, His, Cys, Glu) required for activity (47, 48), can be found in several cosmopolitan marine bacteria. Whether these homologs function in aerobic marine bacteria is questionable, since although one such homolog is present in the genome of *P. inhibens*, it was previously shown to be a methionine auxotroph when *mtgCDE* was mutated (34). However, it is plausible that these are synthesized only under cobalamin limitation, similar to the MetE-fusion protein in eukaryotic microalgae, as an adaptation to cobalamin scarcity (48).

In *R. pomeroyi* DSS-3 and other marine bacteria that encode *mtgC* and *mtgD*, multiple (up to seven) ORFs containing the pfam06253 domain found in MtgB are often found. This suggests that diverse methylated compounds may also be catabolized via this cobalamin-dependent route to serve as energy sources through subsequent oxidation via the H_4_F-linked C1 oxidation pathway (8, 9, 27), expanding the importance of this vitamin in mediating biogeochemical reactions across the ocean (1). In support, anaerobic *Eubacterium* spp. also possess higher numbers of MtgB-like homologs (MtpB, MtbB, MtyB, MtcB, and MthB), which preferentially demethylate proline-betaine, GBT, gamma-butyrobetaine, carnitine, and phosphocholine, respectively, all through an interaction with a single-core cobalamin-binding MtgC subunit (42, 43, 49–51).

In summary, we report the finding of an unexpected connection between GBT catabolism and methionine synthesis in cosmopolitan marine heterotrophs outside of the Pelagibacteraceae family. In addition to uncovering a major route for GBT demethylation in nature, our results expand the known importance of cobalamin across the global ocean.

## Materials and Methods

### Bacterial Growth Conditions.

*R. pomeroyi* DSS-3 was maintained on marine agar 2216 (Difco, UK) or ½ YPSS: yeast extract (YE, 2 g L^−1^), peptone (1.25 g L^−1^), and sea salts (30 g L^−1^, Sigma). Gentamicin (20 μg mL^−1^) was added to maintain the *R. pomeroyi* DSS-3 *ΔmtgC::Gm* mutant, and gentamicin and kanamycin (100 μg mL^−1^) were added to maintain the complemented mutant strain *ΔmtgC::Gm*+*mtgC*:DSS‐3. For all growth experiments, *R. pomeroyi* DSS-3 wild type and mutant strains were grown in a marine ammonium mineral salts (MAMS) medium lacking methionine with the addition of relevant carbon sources. MAMS medium, as modified by (52), contained the following (L^−1^): NaCl, 20 g; (NH_4_)_2_SO_4_, 1 g; MgSO_4_·7H_2_O, 1 g; CaCl_2_·2H_2_O, 0.2 g; FeSO_4_·7H_2_O, 2 mg; Na_2_MoO_4_·2H_2_O, 20 mg; KH_2_PO_4_, 0.36 g; K_2_HPO_4_, 2.34 g plus 1 mL of SL‐10 trace metals solution. Vitamins, including cyanocobalamin 2 μg, were prepared as described previously (53). Either 8 mM succinate, or 5 mM of either GBT, choline, or carnitine was used as the sole carbon source. In these treatments, (NH_4_)_2_SO_4_ remained the source of nitrogen.

### Quantification of Glycine Betaine.

As per our previous studies (8, 9), to quantify GBT, cells were boiled for ≽10 min, cell debris was removed by centrifugation for 5 min at 17,000 × g, and supernatant was analyzed for GBT concentration using a cation-exchange ion chromatograph (881 Compact IC pro, Metrohm, Runcorn, UK) with Metrosep C 4 guard (Metrohm, Switzerland), Metrosep C 4-250/4.0 separation column, and a conductivity detector (Metrohm). An external calibration was used for GBT quantification.

### Proteomics Analysis.

Cells (25 mL) were grown overnight in MAMS medium using either 6 mM succinate, or 5 mM GBT, choline, or carnitine as the sole carbon source. In addition, either 2 mM GBT or ammonium was supplemented as the sole nitrogen source for succinate-grown cells, totaling five treatments, each grown in triplicate. After overnight growth, or when the OD_540_ reached ~0.5 to 0.7, cells were pelleted (10 min at 10,000 × g) and stored at −20°C. After thawing, pellets were resuspended in 3:1 water:LDS sample buffer. Cells were then lysed at 95°C with repeated vortexing. Then, 20 μL of each sample was run on a NuPAGE 10% (wt./vol.) Bis-Tris protein gel for 5 min. The gel was subsequently stained with Coomassie staining and the gel band excised and cut into 1 mm pieces before being destained with 50% (vol./vol.) ethanol and 50 mM ammonium bicarbonate. Gel pieces were dehydrated with pure ethanol, followed by alkylation with 10 mM Tris(2-carboxyethyl)phosphine, 40 mM 2-chloroacetamide, and 50 mM ammonium bicarbonate at 70°C for 5 min. Gel pieces were washed with 50% (vol./vol.) ethanol in 50 mM ammonium bicarbonate twice and dehydrated with pure ethanol. Proteins were then digested with trypsin overnight, and peptides were extracted using repeated 10 min sonication. Peptides were precipitated using a speed vacuum concentrator for 3 h at 45°C and resuspended in 2% acetonitrile with 0.1% trifluoroacetic acid before measurement by high-performance nano ESI liquid chromatography–mass spectrometry as described previously (54). Mass spectrum searches were carried out in MaxQuant and subsequent statistics pipelines were implemented in Perseus as described previously (3). Briefly, label free quantitation (LFQ) (protein-level) based on the precursor ion peak intensity values were log_2_ transformed, with any missing values filled. To determine differential protein synthesis between samples, differences in protein LFQ_log2_ values between the succinate + ammonium control and the four N-osmolyte treatments were calculated.

### Bacterial Genetics.

Previously developed methods were employed to generate the mutant strains in *R. pomeroyi* DSS-3 (8, 55); a full list of strains, plasmids, and primers used to generate the mutants are shown in Dataset S3. The sequence integrity of all constructs was confirmed by DNA sequencing prior to conjugation. The resulting plasmids were transformed into *Escherichia coli* S17.1 via electroporation and mobilized into *R. pomeroyi* DSS-3 via conjugation onto a 0.22-μm pore‐size, 47-mm sterile filter (Millipore, UK), using 1/2 YPSS medium as the medium. Transconjugants were selected on a modified sea salts minimal medium with gentamicin (10 μg mL^−1^) and monomethylamine (3 mM) as the sole nitrogen source as described previously (56). To overcome potential methionine auxotrophy, we supplemented the medium with 0.005% (w/v) yeast extract. After isolation of transconjugants, ½ YPSS medium was used. Double cross‐over mutants were selected by their sensitivity to kanamycin, and homologous recombination was confirmed by PCR and DNA sequencing.

### Bioinformatics.

Genomes related to marine isolates, MAGs, and SAGs in the Integrated Microbial Genomes database (IMG/MER) were scrutinized for ORFs related to cobalamin-dependent methyltransferases and associated GBT catabolism or methionine synthesis. Query sequences used were as follows: SAR11 Bhmt (SAR11_1173); MtgC (*R. pomeroyi* DSS-3, SPO2160; *P. inhibens*, PGA1_c13350); *S. meliloti* Bmt (SMc04325); *R. pomeroyi* MtgB1 (SPO2108); *R. pomeroyi* DSS-3 MtgB2 (SPO2134); *R. pomeroyi* DSS-3 MtgD (SPO1863); *R. pomeroyi* DSS-3 MtgE (SPO1884); *E. coli* MetE (b3829), *S. meliloti* RM1021 MetH (SMc03112). BLASTP was performed using a minimum similarity of 30% and stringency of e-40. Due to the presence of multiple paralogs, the top hit was taken, typically with an e-value of e-130 or less. The “function search” was also used to scrutinize genomes for ORFs containing the associated pfam domains, for example, MetE (pfam01717). To search the TARA oceans dataset, the Ocean Gene Atlas v2 database (57) was scrutinized for the abundance, distribution, diversity and expression of *mtgBCDE*, *bhmt* (SAR11 clade form), and *metH* using profile Hidden Markov Models (pHMM) and a stringency of e-70 or e-80. To generate these pHMMs, sequences (Top hits) retrieved from BLASTP were aligned using MUSCLE, trimmed and phylogenetic reconstruction by maximum likelihood was performed using IQtree2 (58) with parameters -m TESTMERGE -bb 1000 -safe. Trees were visualized using the Integrative Tree of Life (ITOL) online tool (59) and sequences were checked to determine whether they fell within clades containing characterized variants. Sequences obtained from all searches were again aligned using MUSCLE, trimmed and subjected to phylogenetic reconstruction as before. For environmental sequences retrieved from the TARA oceans dataset, clustering was performed using a local version of CD-HIT (cut off 0.9). Taxonomic classification was generated using the Ocean Gene Atlas pipeline. For *mtgBCDE* and *bhmt* gene and transcript abundance, the percentage of mapped reads were generated and normalized against the percentage of mapped reads obtained for 10 single-copy housekeeping genes as per (2, 3).

## Supplementary Material

Appendix 01 (PDF)

Dataset S01 (XLSX)

Dataset S02 (XLSX)

Dataset S03 (XLSX)

## Data Availability

Previously published data were used for this work (57, 60). All other data are included in the manuscript and/or supporting information.
