# Supplementary material for: Methionine synthesis and glycine betaine demethylation are intricately intertwined in cosmopolitan marine bacteria
Source: Proc Natl Acad Sci U S A. 2025 Sep 16;122(38):e2426167122. doi: 10.1073/pnas.2426167122 (PMC12478193; doi:10.1073/pnas.2426167122)
Supplement: Supplementary file 1 — Appendix 01 (PDF) [file pnas.2426167122.sapp.pdf]

Methionine synthesis and glycine betaine demethylation are intricately intertwined in cosmopolitan marine bacteria

Michaela A. Mausz[1], Andrew R.J. Murphy[1], Maria del Mar Aguilo-Ferretjans[1], Andrew Hitchcock[2,3], Mary Ann Moran[4], David J. Scanlan[1], Yin Chen[1,5] and Ian D.E.A. Lidbury[2]*

[1] School of Life Sciences, University of Warwick, Gibbet Hill Road, Coventry, UK

[2] Molecular Microbiology - Biochemistry and Disease, School of Biosciences, University of Sheffield, Sheffield, UK

[3] Plants, Photosynthesis and Soil, School of Biosciences, University of Sheffield, Sheffield, UK

[4] Department of Marine Sciences, University of Georgia, USA

[5] School of Biosciences, University of Birmingham, Edgbaston, UK

*Corresponding author: I.lidbury@sheffield.ac.uk

Table of contents

Supplementary Figures

1. Known pathways of glycine betaine (GBT) demethylation in nature.
2. Phylogeny of the methyltransferase domain (S-methyl-trans - pfam02574) in environmental bacteria.
3. Proteomic assessment of *R. pomeroyi* DSS-3 grown on glycine betaine and other methylated compounds.
4. Cultivation of *R. pomeroyi* DSS-3 strains in a complex medium.
5. Cultivation of *R. pomeroyi* DSS-3 strains in a minimal medium.
6. Gene and transcript abundance profiles for *bhmt* and *mtgBCDE* across the global ocean.
7. The relative contribution of marine bacteria to *mtgC* expression in the ocean.
8. Phylogeny of ORFs containing the pfam01717 domain associated with cobalamin-independent MetE.

Supplementary datasets

1. Comparative proteomic analysis of *Ruegeria pomeroyi* DSS-3 grown on *N*-osmolytes with glucose and ammonium as the control.
2. Comparative genomic analysis of marine bacteria. Genomes were screened for the presence of various glycine betaine catabolic genes as well as methionine synthase (MetH).
3. List of primers used in this study.

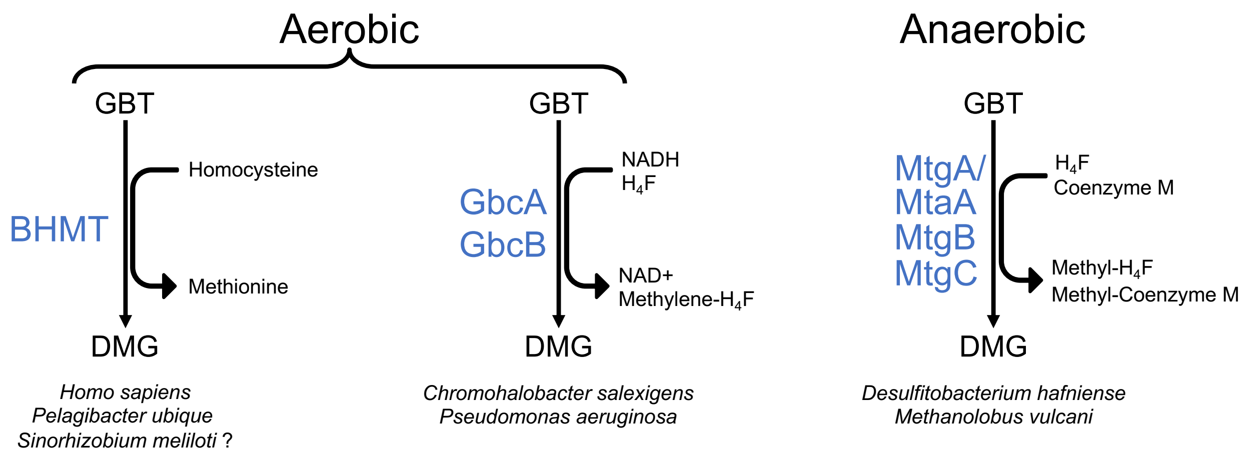

Supplementary Figure 1. Known pathways of glycine betaine (GBT) demethylation in nature. Two aerobic pathways have been identified, though the betaine-homocysteine methyltransferase (BHMT) in *Pelagibacter ubique* and *Sinorhizobium meliloti* has not been experimentally validated. The GBT monooxygenase (GbcAB) has been experimentally validated in two bacterial models. GBT-specific methyltransferases have been characterised in a sulfate-reducing bacterium (*Desulfitobacterium hafniense*) and a methanogenic archaeon (*Methanolobus vulcani*), the former using tetrahydrofolate (H_4F) and the latter using Coenzyme M as the methyl acceptor. Abbreviations; DMG, dimethylglycine.

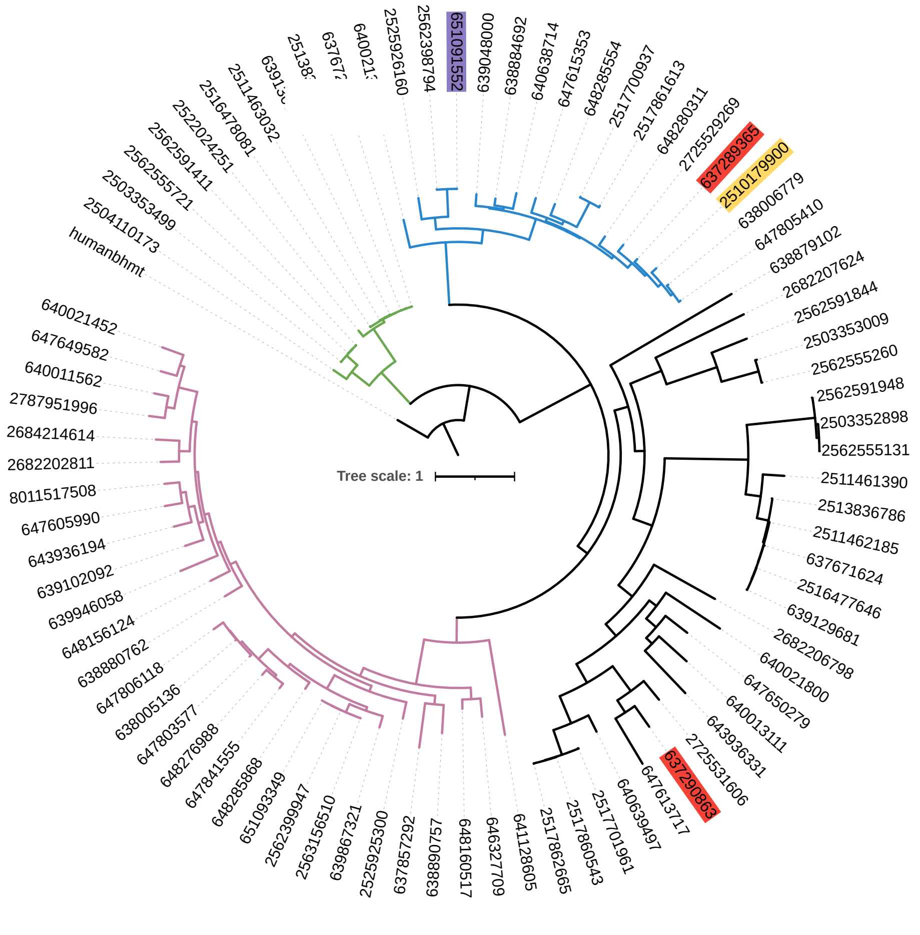

Supplementary Figure 2. Phylogeny of the methyltransferase domain (S-methyl-trans - pfam02574) in environmental bacteria. This protein family harbors the human betaine-homocysteine transferase (set as the outgroup), SAR11 Bhmt (green branches), *S. meliloti* Bhmt (purple), PGA1_c13370 (yellow), which is essential for methionine synthesis, Spo1884 (637289365, red) and Spo3398 (637290863, red). IMG gene accession numbers are provided for each leaf. All domains within the classical MetH have pink branches. Domains related to the split methionine synthase have blue branches.

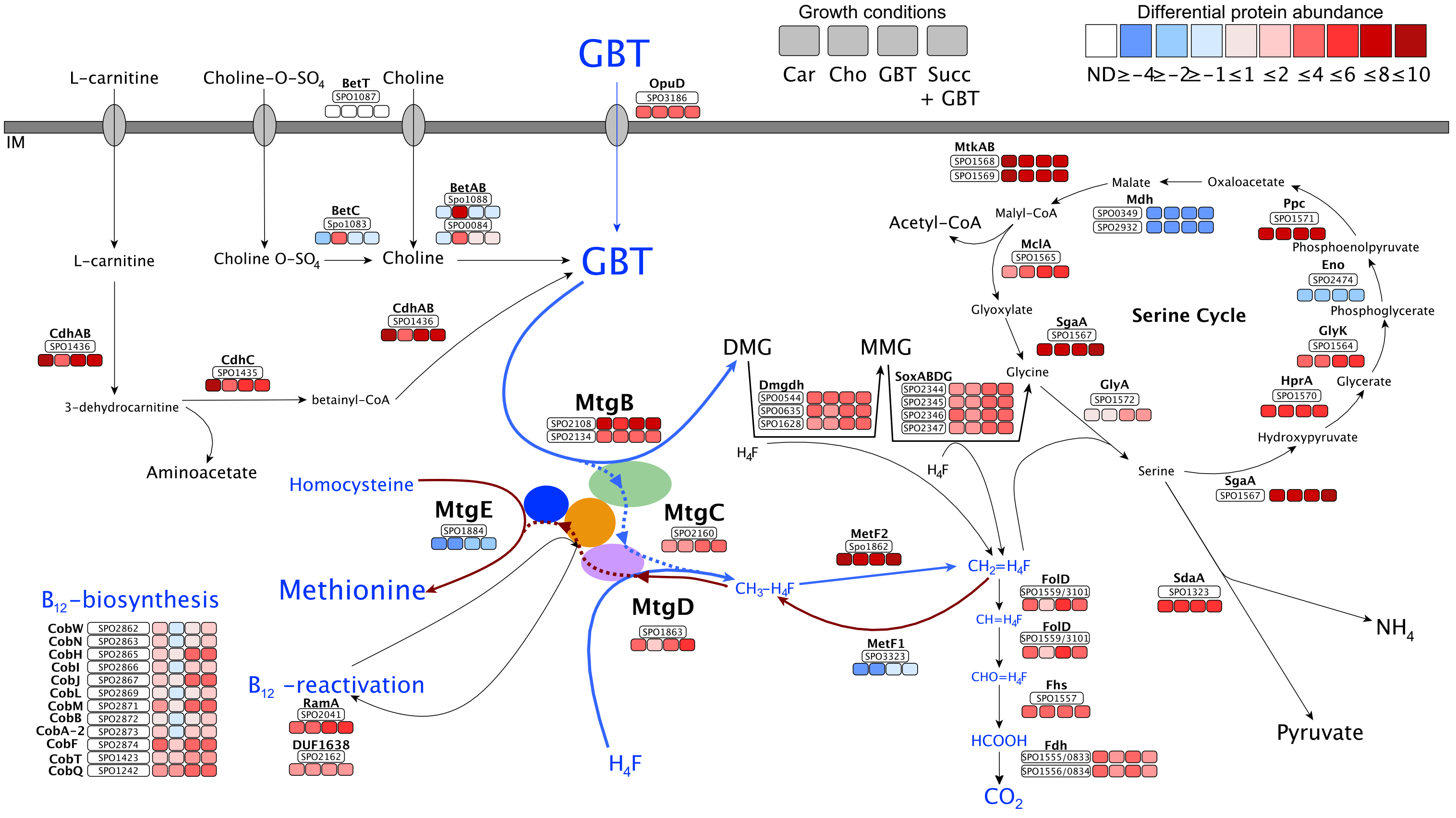

Supplementary Figure 3. Proteomic assessment of *R. pomeroyi* DSS-3 grown on glycine betaine and other methylated compounds. (A) Whole-cell protein extracts were prepared from cells grown on succinate (Succ) and ammonium (control) or GBT, choline (Cho) or carnitine (Car) as the sole carbon source. A fifth treatment with Succ as the carbon source and GBT as the sole nitrogen source was also analysed. MtgB (green) donates a methyl group to MtgC: cobalamin (orange) and MtgD (purple) transfers the methyl group onto tetrahydrofolate (H4F), depicted by a black dotted line. We hypothesise MtgD also works in reverse (red dotted line) to transfer the methyl group to MtgC:cobalamin followed by transfer to MtgE (blue), which converts homocysteine to methionine. The mean (n=3) difference in LFQLog2 values between control and N-osmolyte treatments is presented. IM, inner membrane; ND, not detected.

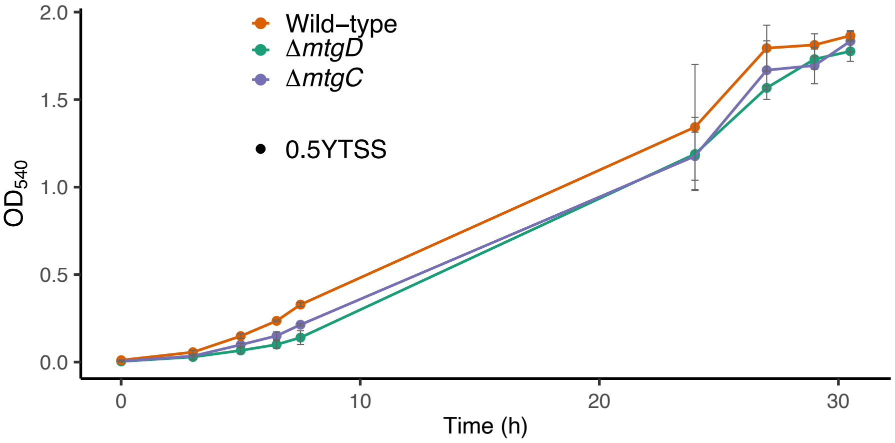

Supplementary Figure 4. Cultivation of *R. pomeroyi* DSS-3 strains in a complex medium. The wild-type and two mutants (Δ*mtgC*) and (Δ*mtgD*) were grown in ½ YPSS. Results are the mean of triplicate cultures and error bars denote standard deviation.

A)

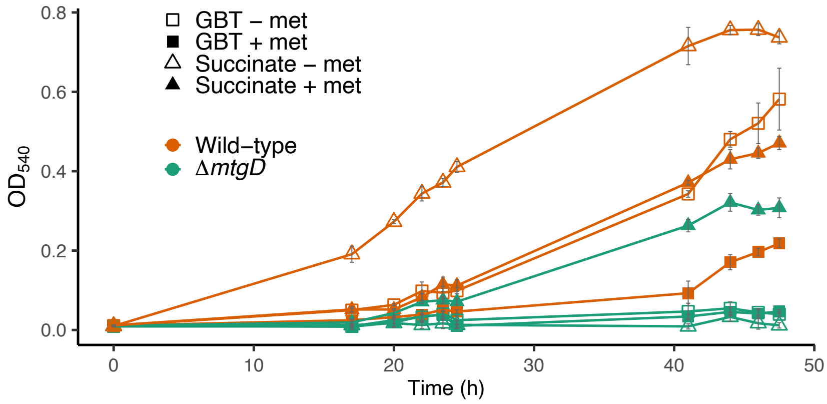

B)

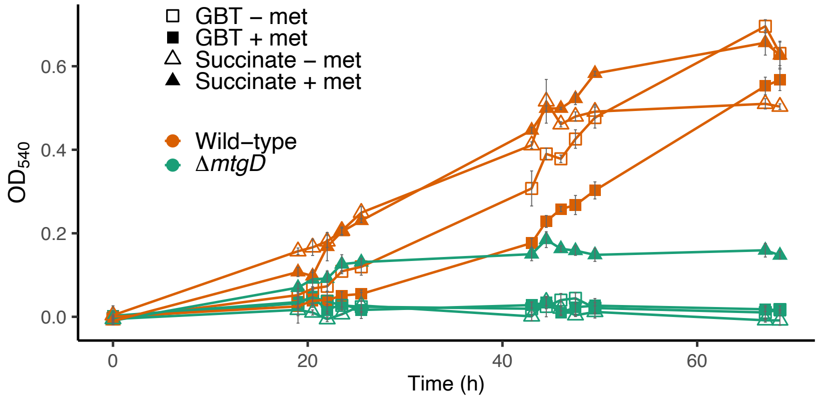

Supplementary Figure 5. Cultivation of *R. pomeroyi* DSS-3 strains in minimal medium. Wild-type and mutant (Δ*mtgD*) were grown using either glycine betaine (GBT) or succinate as the sole carbon source, in the absence or presence of methionine. Either 100 µM (A) or 20 µM (B) methionine was used. Results demonstrate MtgD is essential for both methionine synthesis and glycine betaine catabolism. Results are the mean of triplicate cultures and error bars denote standard deviation.

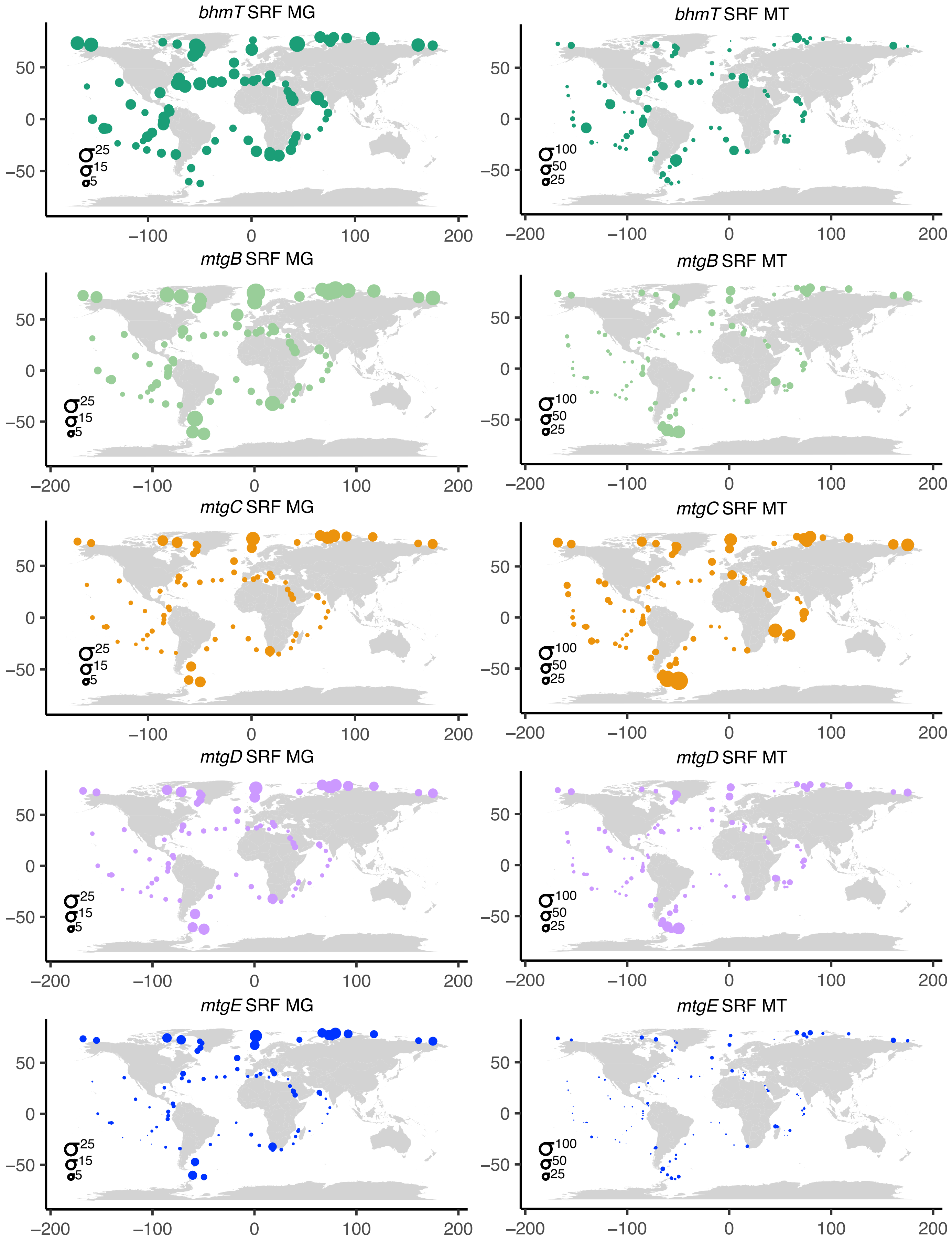

Supplementary Figure 6. Gene and transcript abundance profiles for *bhmt* and *mtgBCDE* across the global ocean. Data was extracted from the TARA oceans dataset, using the Ocean Gene Atlas portal. The geographical location of each site is depicted on the map. SRF = surface. MG = metagenome. MT = metatranscriptome. Dot sizes represent the abundance of each gene/transcript normalised against a set of core housekeeping genes. Values depict genome equivalent (%).

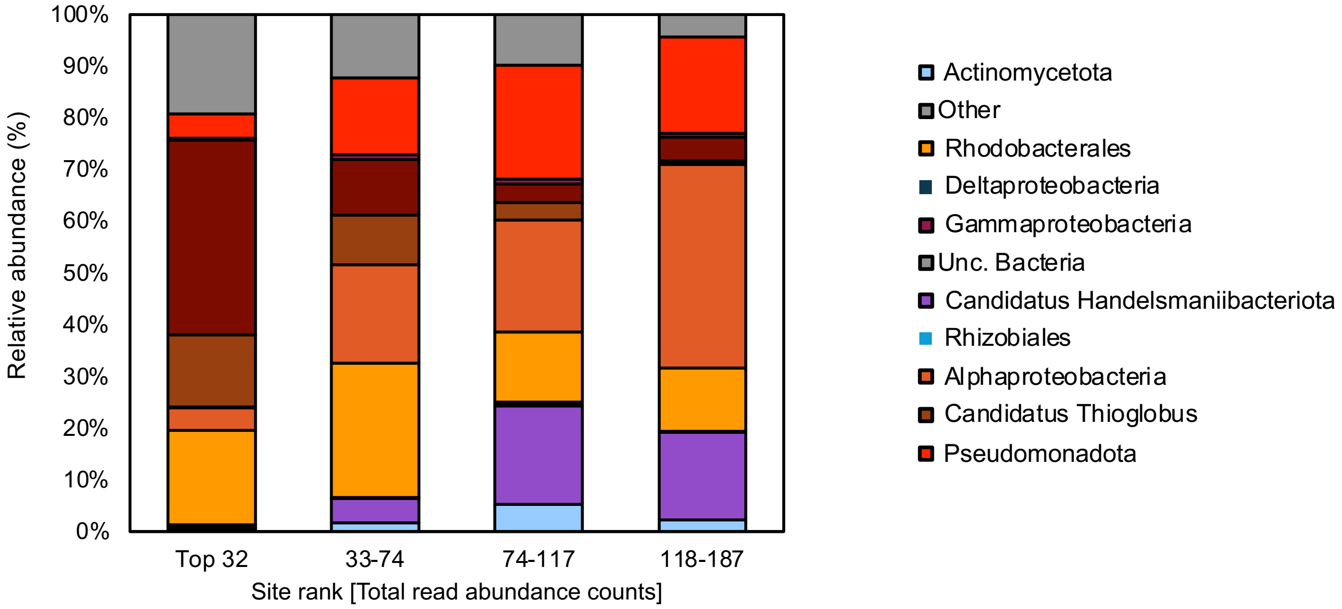

Supplementary Figure 7. The relative contribution of marine bacteria to *mtgC* expression in the ocean. TARA Oceans sampling sites were separated and grouped based on their overall rank according to total *mtgC* expression levels. Within each grouping the relative contribution (%) of each taxonomic group was calculated.

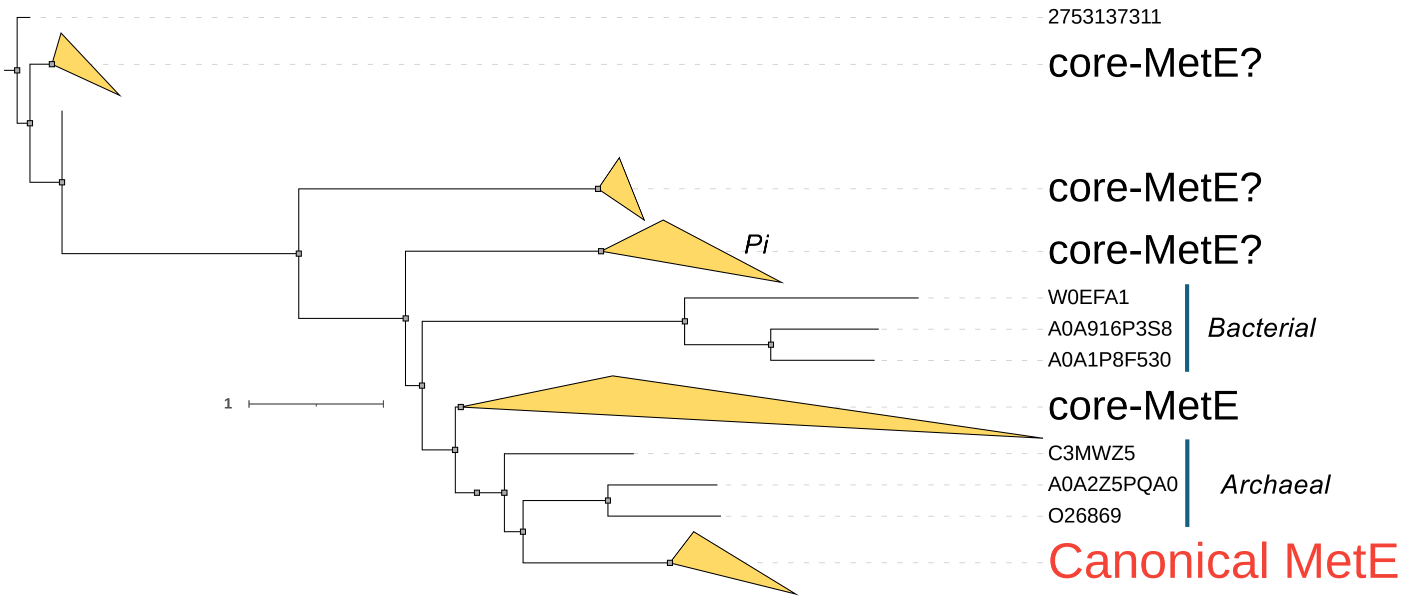

Supplementary Figure 8. Phylogeny of ORFs containing the pfam01717 domain associated with cobalamin-independent MetE. Genomes from supplementary table 2 were searched using the pfam domain in IMG/JGI. In addition, various Bacteroidota were added the the search. Only the core catalytic domain was used to reconstruct the evolutionary distances. The maximum-likelihood tree contains 402 sequences, of which all (with very few exceptions) possessed the four catalytic residues (Cys_2, His, Glu) that coordinate Zn^{2+} binding. The characterised archaeal and bacterial core-MetE were also included and their Uniprot identifiers are provided. The clade containing the putative core-MetE homolog identified in *P. inhibens* is labelled (*Pi*). Under the conditions tested in Price et al. (2018), the *Pi* homolog did not function as a methionine synthase when components of the split methionine synthase were mutated.
